# Looking inside the cell

**DOI:** 10.7554/eLife.33650

**Published:** 2017-12-05

**Authors:** Eric C Arakel, Blanche Schwappach

**Affiliations:** Department of Molecular BiologyUniversity Medical Center GöttingenGöttingenGermany

**Keywords:** cryo-electron tomography, membrane trafficking, Chlamydomonas reinhardtii, COPI, subtomogram averaging, vesicles, Other

## Abstract

Advances in imaging techniques have shed new light on the structure of vesicles formed by COPI protein complexes.

**Related research article** Bykov YS, Schaffer M, Dodonova SO, Albert S, Plitzko JM, Baumeister W, Engel BD, Briggs JAG. 2017. The structure of the COPI coat determined within the cell. *eLife* **6**:e32493. doi: 10.7554/eLife.32493

Vesicles perform a wide range of functions within cells, such as the transport of proteins and lipids between the different parts of a cell. Each vesicle is coated with a protein complex, and understanding the structure and function of these complexes is a central challenge in cell biology. Two of these complexes – COPII and clathrin – are relatively well understood, but the third, which is called COPI, is not.

Vesicles coated with COPI are responsible for transport from the Golgi to the endoplasmic reticulum, and also for transport between the cisternae within the Golgi (Beck et al., 2009). The COPI complex contains seven subunits that are recruited *en bloc* to the vesicle membrane (Hara-Kuge et al., 1994). However, the intricate couplings between these subunits have long hindered efforts to determine the molecular architecture of the COPI coats.

The basic model of COPI vesicle formation begins with the recruitment of a GTPase called Arf1 to a membrane. Arf1 is then loaded with GTP, thereby activating it. COPI complexes then interact with the GTP-loaded Arf1 and cargo proteins to form the vesicle, which can then detach from the membrane and transport the cargo proteins to their destination. Finally, the coat disassembles in a process that is called un-coating: three GTPase activating proteins (ArfGAP1/2/3) play an important role in this process by stimulating the hydrolysis of the GTP and, therefore, deactivating Arf1 (Lanoix et al., 1999; Pepperkok et al., 2000; Weimer et al., 2008). Subsequent studies of this un-coating process suggested that Arf1 and COPI disengage from membranes independently, in a non-cooperative manner (Presley et al., 2002; Yang et al., 2002). These and other results led to a model in which the individual COPI complexes were held in place by lateral linkages within the coat and by interactions with the underlying cargo.

These studies were built upon in a series of in-vitro reconstitution experiments undertaken by a collaboration between the groups of Felix Wieland (University of Heidelberg) and John Briggs (European Molecular Biology Laboratory and the MRC Laboratory of Molecular Biology). The in-vitro reconstitution approach involves combining purified proteins and liposomes (a type of vesicle) in a test tube to generate COPI-coated vesicles, thus allowing researchers to study the precise sequence of events that take place during vesicle formation. Wieland, Briggs and co-workers exploited significant advances in two techniques – cryo-electron tomography and subtomogram averaging – to determine the molecular structure of the COPI coat (Dodonova et al., 2017; Dodonova et al., 2015; Faini et al., 2012).

However, the process of vesicle formation in these seminal experiments differed from the process in real cells in a number of ways: the vesicles did not contain any cargo, and the Arf1 was frozen in its activated state because the researchers used a variant of GTP called GTPγS that cannot be hydrolysed. This meant that several questions remained unanswered: Does early GTP hydrolysis contribute to a pronounced conformational change of the coat (such as the exposure of cargo-recognition sites)? Are the linkages within the coat rearranged to accommodate cargo? How does Arf1 disengage from COPI without perturbing the pre-existing lattice? And how does the cage adapt structurally to the loss of Arf1?

The rational next step would have involved using GTP (rather than GTPγS), the three ArfGAPs and cargo proteins in in-vitro reconstitution experiments, which would have been very arduous. However, in an unexpected development reported in eLife, Briggs, Benjamin Engel and Wolfgang Baumeister (both of the Max Planck Institute of Biochemistry) and co-workers – including Yury Bykov as first author – describe the native structure of COPI-coated vesicles in a species of green algae called *Chlamydomonas reinhardtii* (Bykov et al., 2017). This quantum leap was made possible through a combination of cryo-focused ion-beam milling (a technique that involves vitrifying cells and then using a focused ion beam to slice off ultrathin sections) and further improvements in cryo-electron tomography and subtomogram averaging. Moreover, this approach meant that the researchers were able to study the structure of the coat in the presence of active Arf1, the relevant ArfGAPs and the vesicle's cargo.

Building on their previous systematic characterization of the morphology of the Golgi in *C. reinhardtii* (Engel et al., 2015a; Engel et al., 2015b), and what is known about the structures of COPI, COPII and clathrin, Bykov et al. were able to identify the three different types of coated vesicles in the vicinity of the cisternae within the Golgi. The researchers were also able to determine the in-situ structure of the coat without relying on existing structural models, and found that it was remarkably similar to the structure that emerged from the in-vitro reconstitution experiments. This finding is significant for the field because it strongly suggests that the existing structural model is physiologically relevant.

Moreover, an additional density was observed inside the vesicles in *C. reinhardtii* that was not visible in the in-vitro experiments. This density is likely to correspond to cargo, and the fact that it was observed under the β-propeller of the β’-subunit, but not beneath the β-propeller of the α-subunit (which is structurally similar), suggests that this cargo is recognized by a specific subunit of COPI. It is also striking that this density is seen in many vesicles adjoining both the cis and medial cisternae.

Remarkably, the relative proportions of Arf1 and COPI were the same for most vesicles, irrespective of the intactness of the coat: this suggests that, upon hydrolysis, the constituents of the coat are all released at the same time to allow the vesicle to fuse with an acceptor compartment, and argues against a step-wise dissociation of the coat from the vesicle.

In addition to demonstrating the ability of in-situ experiments to distinguish between models based on in-vitro experiments, the work of Bykov et al. highlights the ability of new techniques to allow us to see things – such as the density under the β-propeller of the β’-subunit – that we have missed so far. We are keen to get a glimpse of COPII and clathrin in situ too.
